# PET/MRI in head and neck cancer: initial experience

**DOI:** 10.1007/s00259-012-2248-z

**Published:** 2012-09-28

**Authors:** Ivan Platzek, Bettina Beuthien-Baumann, Matthias Schneider, Volker Gudziol, Jens Langner, Georg Schramm, Michael Laniado, Jörg Kotzerke, Jörg van den Hoff

**Affiliations:** 1 Department of Radiology, Dresden University Hospital, Fetscherstr. 74, 01307 Dresden, Germany; 2 Department of Nuclear Medicine, Dresden University Hospital, Dresden, Germany; 3 Oral and Maxillofacial Surgery, Dresden University Hospital, Dresden, Germany; 4 Department of Otolaryngology, Dresden University Hospital, Dresden, Germany; 5 Helmholtz-Zentrum Dresden-Rossendorf, Institute of Bioinorganic and Radiopharmaceutical Chemistry, Dresden, Germany; 6 Nuclear Medicine, Dresden University Hospital, Dresden, Germany

**Keywords:** PET/MRI, Head and neck cancer

## Abstract

**Purpose:**

To evaluate the feasibility of PET/MRI (positron emission tomography/magnetic resonance imaging) with FDG (^18^F-fluorodeoxyglucose) for initial staging of head and neck cancer.

**Methods:**

The study group comprised 20 patients (16 men, 4 women) aged between 52 and 81 years (median 64 years) with histologically proven squamous cell carcinoma of the head and neck region. The patients underwent a PET scan on a conventional scanner and a subsequent PET/MRI examination on a whole-body hybrid system. FDG was administered intravenously prior to the conventional PET scan (267–395 MBq FDG, 348 MBq on average). The maximum standardized uptake values (SUV_max_) of the tumour and of both cerebellar hemispheres were determined for both PET datasets. The numbers of lymph nodes with increased FDG uptake were compared between the two PET datasets.

**Results:**

No MRI-induced artefacts where observed in the PET images. The tumour was detected by PET/MRI in 17 of the 20 patients, by PET in 16 and by MRI in 14. The PET/MRI examination yielded significantly higher SUV_max_ than the conventional PET scanner for both the tumour (*p* < 0.0001) and the cerebellum (*p* = 0.0009). The number of lymph nodes with increased FDG uptake detected using the PET dataset from the PET/MRI system was significantly higher the number detected by the stand-alone PET system (64 vs. 39, *p* = 0.001).

**Conclusion:**

The current study demonstrated that PET/MRI of the whole head and neck region is feasible with a whole-body PET/MRI system without impairment of PET or MR image quality.

## Introduction

Head and neck cancer ranks among the ten most common malignant diseases [1]. The vast majority of head and neck malignancies are squamous cell carcinomas [2]. The choice of therapy in patients with head and neck cancer depends mainly on tumour location, the invasion of adjacent structures and on the presence of metastases [3]. MRI provides excellent soft tissue contrast, which is useful for differentiating masses from neighbouring tissues and has facilitated the widespread use of MRI for head and neck imaging. MRI is considered the modality of choice for imaging tumours of the oral cavity [4] and the pharynx [5]. However, the sensitivity of MRI for metastatic lymph node disease is rather low, as it relies on morphologic criteria for lymph node evaluation [6]. ^18^F-Fluorodeoxyglucose (FDG) PET has superior sensitivity for the detection of cervical lymph node metastases in comparison with both CT and MRI [7, 8]. The recently introduced whole-body PET/MRI systems [9] combine the unique metabolic imaging capabilities of PET with the superb soft tissue contrast of MRI. PET/MRI thus appears to be a promising modality for the imaging of head and neck malignancies. The aim of this pilot study was to evaluate the feasibility of FDG-PET/MRI for initial staging of head and neck cancer.

## Materials and methods

### Patients

The study was approved by the local ethics committee and all patients gave written informed consent before being included. The study group comprised 20 patients (16 men, 4 women) aged between 52 and 81 years (median age 64 years) with histologically proven squamous cell carcinoma of the head and neck region. The patients were examined using both a stand-alone PET scanner and a whole-body PET/MRI system. Tumour locations included the floor of the mouth (six patients), tongue (five), mandible (three), lower lip (two), maxillary sinus (one), piriform sinus (one), maxilla (one) and the palatine tonsils (one).

### PET

The patients were instructed to refrain from food intake for at least 6 h before FDG injection, while fluid intake (water or tea without sugar) was encouraged. PET imaging was performed with a dedicated PET scanner (ECAT EXACT HR+; Siemens, Erlangen, Germany; axial field of view of 15.25 cm, reconstructed isotropic spatial resolution about 6.5 mm). The scanner’s septa allowed examinations in either 2D or 3D mode. Prior to the examination, 4.5 MBq ^18^F-FDG/kg body weight was administered intravenously (267–395 MBq ^18^F-FDG per patient, 348 MBq on average; in-house production) [10]. The time between tracer administration and the start of the PET scan varied between 53 and 110 min (average 64 min). Emission and transmission scanning (^68^Ge/^68^Ga rod sources) covered the body from the proximal femora to the skull base. The scan was performed with the patient in the supine position, arms down at the sides. Eleven patients were examined in 3D mode and nine patients in 2D mode. Transmission time per bed position was 4 min for both the 2D and 3D scans, while emission time per bed position was 8 min for the 2D scans and 4 min for the 3D scans. Overlap between bed positions was 1 cm for the 2D scans and 1.5 cm for the 3D scans. The effective axial field of view was 13.75 cm in 2D mode and 12.75 cm in 3D mode.

### PET/MRI

After the first PET scan the patient was transferred to the adjacent PET/MRI scanner (Ingenuity TOF PET/MRI scanner; Philips Medical Systems, Cleveland, OH). The PET component of the system features time-of-flight technology, an axial field of view of 18 cm, 9 cm overlap between bed positions and a reconstructed isotropic spatial resolution of about 5.5 mm. The patients were examined in the supine position, with arms down at the sides. The PET/MRI examination consisted of a low-resolution nondiagnostic attenuation MR scan of the head, neck and thorax, followed by a PET scan and a diagnostic MR scan of the head and neck.

The attenuation MR scan is a nondiagnostic T1-weighted fast field echo scan used for PET attenuation correction. It covers the head, neck and thorax following the manufacturer‘s recommendations. Although the patient is positioned in a 16-channel phased array neurovascular coil from the start of the PET/MRI examination, attenuation MR scans are acquired with the integrated quadrature body coil. An MR-based attenuation map used for attenuation correction in PET image reconstruction is created via segmentation of the attenuation MR image volume into three tissue classes (air, lung and soft tissue) followed by an assignment of respective attenuation values (0, 0.022 and 0.096 cm^-1^) [11, 12]. The effects of the phased array coil on PET attenuation are taken into account via a corresponding vendor-provided attenuation template, which is included in the final attenuation image.

The average time between tracer injection and the start of the second PET scan was 177 min (143–225 min). Three bed positions were used to cover the complete head and neck region. Emission time was 6 min for each bed position. The position of the patient on the scanner table remained unchanged during the whole examination in order to achieve optimal coregistration of the PET and MR data.

Total imaging time for the PET/MRI examination was 39 min, including 18 min for the PET scan.

Diagnostic MR images were acquired with a 16-channel phased array neurovascular coil. The diagnostic MR scan included short tau inversion recovery (STIR) turbo spin echo (TSE) images in axial and coronal orientation, T1-weighted TSE images in axial orientation, T1-weighted contrast-enhanced TSE images with fat saturation in axial orientation and T1-weighted contrast-enhanced turbo field echo images with fat suppression in coronal orientation. Sequence parameters are summarized in Table 1. Gd-DTPA (0.2 ml/kg body weight; Magnevist®. Bayer Schering Pharma, Berlin, Germany) was injected intravenously, followed by 20 ml saline. Fused PET/MR images were produced using the Philips Fusion Viewer software.

**Table 1:** Sequence parameters

Sequence	Orientation	Field of view (mm)	Number of slices	Slice thickness (mm)	TR (ms)	TE (ms)	Matrix	Acquisition time
STIR_long TE cor	Coronal	250 × 199	30	3	4354	60	512	4 min 47 s
STIR_long TE tra	Axial	250 × 180	30	4	4354	60	512	4 min 21 s
T1W_TSE tra	Axial	250 × 159	24	4	450	9.2	512	3 min 50 s
THRIVE	Coronal	250 × 199	150	2	7.5	3.6	256	3 min 17 s
T1_SPIR CE	Axial	250 × 159	25	4	656	9.2	512	5 min 31 s

*FOV* field of view; *TR* repetition time; *TE* echo time; *STIR* short-tau inversion recovery; *TSE* turbo spin echo; *THRIVE* T1-weighted high-resolution isotropic volume acquisition; *SPIR* spectral presaturation with inversion recovery; *CE* contrast enhanced

### Image interpretation

MR images were evaluated on a Philips Extended MR Workspace (EWS) console by two board-certified radiologists without access to the PET data. In the event of differing results, the final decision was made in consensus. Cervical lymph nodes with the shortest diameter more than 10 mm were considered malignant [13]. Further imaging findings regarded as suggestive for malignant lymph node disease were a spherical lymph node shape [14], nodal necrosis [15] and irregular lymph node borders [16]. PET scans were visually evaluated by two board-certified nuclear medicine physicians without access to the MRI data. Lesions that appeared to have increased tracer uptake in comparison to the salivary glands and the muscles were considered malignant [17]. In the event of differing results, the final decision was made in consensus. PET/MR images were evaluated by a board-certified radiologist and a board-certified nuclear medicine physician in consensus.

Both PET datasets were compared visually with a focus on tumour delineation, the conspicuity of lymph node metastases and artefacts. The maximum standardized uptake values (SUV_max_) of the tumour and of both cerebellar hemispheres were determined for both PET datasets using the ROVER® software package (ABX advanced biochemical compounds, Radeberg, Germany). The software allows the semiautomatic analysis of 3D regions of interest, providing parameters including metabolically active volume and SUV_max_ [18].

### Statistical analysis

The Wilcoxon matched-pairs test was used to compare the conventional PET scanner and the PET/MRI system with regard to the number of lymph nodes with increased FDG uptake, and the SUVs of the tumour and the cerebellum. Cohen’s kappa was used to evaluate interrater agreement for primary tumour detection. In contrast, kappa with linear weighting was used to assess interrater agreement for lymph nodes with increased FDG uptake, as in this case a different number of detected lymph nodes is not equal to complete disagreement between the readers. Data were analysed using MedCalc 12.0 (MedCalc Software bvba, Mariakerke, Belgium). A *p* value <0.05 was considered statistically significant.

## Results

**Fig. 1:**
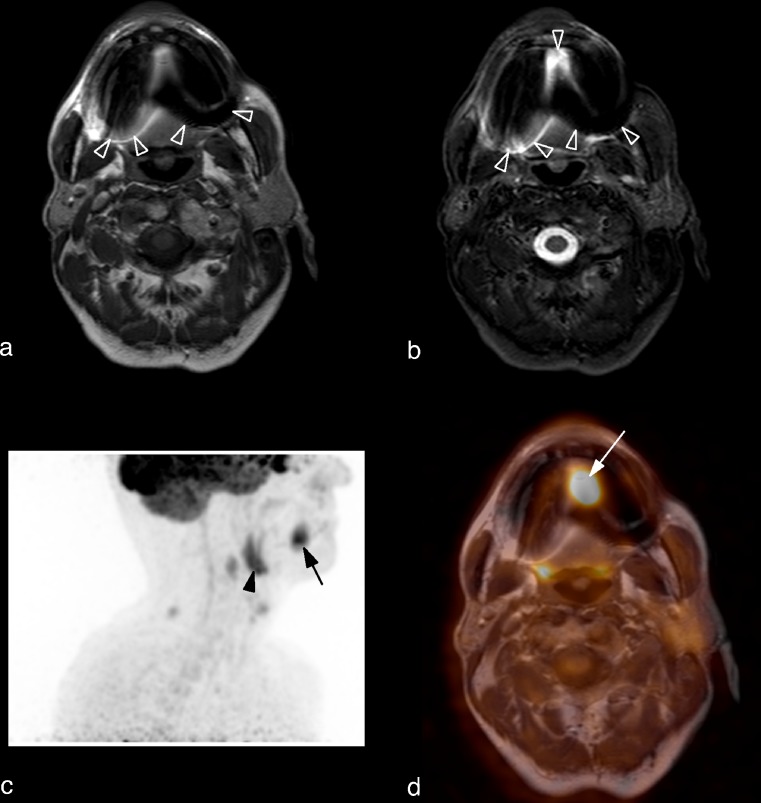
Squamous cell carcinoma of the tongue in a 55-year-old man. **a** Axial T1-weighted TSE image. The tumour is not detectable due to susceptibility artefacts caused by dental implants (*arrowheads*). **b** Axial STIR TSE image showing nearly identical susceptibility artefacts (*arrowheads*). **c** Maximum intensity projection image of the PET dataset showing the tumour (*arrow*) and a lymph node metastasis (*arrowhead*). **d** Axial fused PET/MR image. The tumour is clearly visible (*arrow*)

The primary tumour was detected by PET/MRI in 17 of the 20 patients, by PET in 16 and by MRI in 14 (Fig. 1). Both nuclear medicine physicians evaluating the PET data detected 16 tumours on both PET datasets. Thus the resulting kappa was 1.0 in both cases. One radiologist identified 13 tumours on the MR images, while the second reader identified 14 tumours on the MR images (interrater agreement κ = 0.89).

**Fig. 2:**
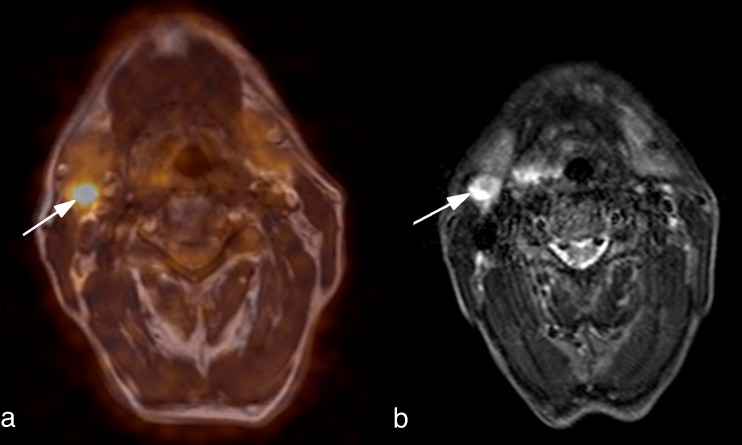
Histologically proven lymph node metastasis in a 60-year-old man with a squamous cell carcinoma of the right piriform sinus. **a** Axial fused PET/MR image. The metastatic lymph node is easily recognizable due to increased FDG uptake (*arrow*). **b** Axial STIR TSE image. In this patient the metastasis was also detected by MRI alone (*arrow*)

Lymph nodes suspicious for metastatic disease were seen in 13 patients (Fig. 2).

Using the stand-alone PET scanner, 39 cervical lymph nodes with increased FDG uptake were detected visually, and using the PET datasets from the PET/MRI system, 64 were detected. The calculated interrater reliabilities were κ = 0.91 for the images acquired with the conventional system (39 vs. 35 lymph nodes with increased FDG uptake) and κ = 0.93 for the PET images acquired with the hybrid system (64 vs. 66 lymph nodes with increased FDG uptake). The numbers of lymph nodes detected using the two PET datasets differed significantly (Wilcoxon test, *p* = 0.001). No MRI-induced artefacts were observed in the PET images from the PET/MR system. There was also no evidence for MR image artifacts caused by the PET hardware of the PET/MR system.

SUV_max_ was evaluated in 16 patients, as in the remaining 4 patients the primary tumour was not detected by PET. The PET/MRI scans yielded a mean SUV_max_ of 14.3 for the tumours (95% CI 11.3 to 17.3), while the conventional PET scans yielded a mean SUV_max_ of 10.0 (95% CI 7.4 to 12.6) for the same lesions. The mean SUV_max_ of the cerebellum was 10.4 for the PET/MRI scans (95% CI 9.00 to 11.8) and 8.9 for the conventional PET scans (95% CI 8.0 to 9.9). The SUV_max_ values from the PET/MR scans were significantly higher than those from the conventional PET scans for both tumour (*p* < 0.0001) and cerebellum (*p* = 0.0009).

Tumour resection was performed in 13 patients, laser excision in 2, laryngectomy in 1, primary radiotherapy in 2 and palliative systemic therapy in 1. In one patient, the PET/MRI scan revealed a mass in the lower lobe of the right lung, which was highly suspicious for malignancy. Histologic samples obtained by bronchoscopy showed a bronchial carcinoma, while a CT scan of the lung showed additional metastases in both lungs. Another patient had a small pulmonary node in the upper lobe of the right lung, which had strong FDG uptake and was rated as a metastasis. In this patient, a partial resection of the upper lobe was performed. The nodule turned out to be an aspergilloma. The patient was treated by tumour resection and neck dissection.

## Discussion

Staging of head and neck cancer requires imaging of the whole head and neck region. The current study demonstrated that comprehensive PET/MR of the head and neck region is feasible with a whole-body PET/MR system without impairment of PET or MR image quality. Early PET/MR systems were essentially restricted to brain examinations because of the limited axial field of view of the available PET inserts. The feasibility of PET/MRI of the head and upper neck using this scanner type in patients with nasopharyngeal carcinoma was previously demonstrated [19]. However, the imaging of the head and upper neck only is insufficient in patients with malignancies of the oral cavity, oro- and hypopharynx and larynx. In these patients, either the primary tumour (e.g. carcinoma in the hypopharynx or larynx) or cervical lymph node metastases of the lower neck will not be detected. In contrast, the current study demonstrated the feasibility of an imaging protocol that allows evaluation of the whole head and neck region, in an analogous manner to MRI scans used in clinical routine.

As PET/MR is a new modality, data concerning image quality of PET/MRI scans are scarce. Promising results regarding PET image quality have already been reported by Boss et al. for PET/MRI examinations of the brain [20]. These authors described streak artefacts on PET images, which are related to the design of early simultaneous PET/MRI systems, and can be reduced by filtering. In the current study, no such artefacts were observed. The improved signal-to-noise-ratio achieved with time-of-flight PET is a significant advantage of the sequential scanner design. While signal-to-noise-ratio improvement is most pronounced in the imaging of abdominal lesions, it has also been documented for other body parts, including the neck [21]. Time-of-flight PET is currently not feasible with simultaneous PET/MR systems because of limitations of the avalanche photodiodes used [22]. In contrast, sequential designs utilize photomultiplier tubes which allow full advantage to be taken of the excellent timing behaviour of state-of-the-art scintillator crystals such as LYSO [23].

Longer acquisition times are a potential disadvantage of sequential PET/MRI systems in comparison to simultaneous PET/MR systems. While this might be a significant factor in brain examinations that include dynamic PET measurements of substantial duration, the increase in scan time for the head and neck region is much less pronounced. In the present study PET was responsible for 46% of the total acquisition time (18 min out of 39 min). The choice of MR sequences has a large impact on PET/MRI scan time. In our study the MRI protocol was nearly identical to the standard head/neck MRI protocol used in our hospital. More clinical data are needed to decide if some MRI sequences can be omitted from the PET/MRI protocol and thus reduce scan time.

Significantly higher tumour SUV_(max)_ values were found with the PET/MRI scans than with the first PET scans (60 min after injection). Although the comparison of SUV values determined with different scanner types is problematic, the leading factor for this effect can be attributed to tumour pathophysiology. Tumour uptake of FDG increases with time, leading to higher SUV_max_ values at later time points [24]. The increase in cerebellar SUV_max_ values was less pronounced, possibly due to an already physiologically decreasing FDG uptake of brain tissue at later time points [25]**. ** Another possible factor causing SUV differences may be the attenuation correction methods used in conventional PET and PET/MRI. Comparing PET and PET/CT, Nakamoto et al. [26] found slightly higher radioactive concentrations for CT-corrected emission images than for Ge-corrected images. Later studies, however, did not find significant differences between SUVs calculated with PET and PET/CT [27]. Currently, the influence of MRI-based attenuation correction on SUVs is being studied and its magnitude has not been clarified.

The number of lymph nodes with increased FDG uptake detected using the PET dataset from the hybrid system was significantly higher than the number from the stand-alone PET scanner. This observation can be explained mainly by a combination of two beneficial effects. First, the stand-alone PET scans were performed earlier than the PET/MRI scans (64 min vs. about 177 min after injection on average). An extended FDG uptake period before the PET/MRI scan leads to an increased contrast of FDG-avid structures relative to the surrounding normal tissue since the contribution of free FDG in tissue to the image signal is reduced at later times. Moreover, the spatial resolution of the PET images delivered by PET/MRI systems is superior to that delivered by stand-alone PET systems, because of reduced partial volume effects and signal loss in small structures. Both factors increase detectability of small lesions by maintaining a sufficient target-to-background contrast.

While PET/MRI is not intended to be a replacement for PET/CT, it has the potential to improve diagnostic imaging in patients in whom the soft tissue contrast provided by CT is deemed insufficient, for example in head and neck or pelvic tumours. Furthermore, PET/MRI has the potential to significantly reduce radiation exposure in comparison to PET/CT. Further studies are needed to evaluate the clinical role of PET/MRI in head and neck cancer.
